# Predictive Factors of Anxiety and Depression in Patients with Type 2 Diabetes Mellitus

**DOI:** 10.3390/jcm13103006

**Published:** 2024-05-20

**Authors:** Oana Albai, Bogdan Timar, Adina Braha, Romulus Timar

**Affiliations:** 1 Department of Second Internal Medicine Diabetes, Nutrition, Metabolic Diseases, and Systemic Rheumatology, “Victor Babes” University of Medicine and Pharmacy, 300041 Timisoara, Romania; albai.oana@umft.ro (O.A.); braha.adina@umft.ro (A.B.); timar.romulus@umft.ro (R.T.); 2 Department of Diabetes, Nutrition and Metabolic Diseases Clinic, “Pius Brînzeu” Emergency Clinical County University Hospital, 300723 Timisoara, Romania; 3 Centre for Molecular Research in Nephrology and Vascular Disease/MOL-NEPHRO-VASC, “Victor Babes” University of Medicine and Pharmacy, 300041 Timisoara, Romania

**Keywords:** type 2 diabetes mellitus, glycemic control, depression, anxiety, psychiatric disorders, quality of life

## Abstract

**Background**: Diabetes mellitus (DM) is a chronic condition associated with multiple complications and comorbidities. Some of these comorbidities are anxiety and depression, with a negative impact on the quality of life, non-adherence to treatment, and poor prognosis. The main **aim** of this study was to evaluate depression and anxiety in a group of patients with DM and their impact on quality of life and identify factors that improve the prognosis and increase the life expectancy and quality of life of patients with DM. **Methods**: A total of 209 patients with type 2 DM (T2DM) were enrolled cross-sectionally. Patients were screened for psychiatric disorders, cognitive impairment, and metabolic parameters. **Results**: Included patients had a median age of 66.0 (58; 70) years, a median DM duration of 9 (6; 15) years, and a suboptimal glycemic control reflected by a median HbA1c of 7.8 (7; 9.2) mg/dL. Patients presented anxiety at different stages in 51.5% of cases, and similarly, depression in 37.5% of cases. Age, duration of DM, HbA1c, and postprandial hyperglycemia (PPG) were predictive factors for anxiety and depression in patients with T2DM. An age > 57 years (sensitivity 84.3, specificity 33.7, AUROC 0.621, *p* = 0.002) and an HbA1c > 8.5% (sensitivity 45.8, specificity 83.1, AUROC = 0.635, *p* < 0.0001) predict a higher rate of anxiety, respectively, of depression in these patients. **Conclusions**: Patients with T2DM have an increased rate of anxiety and depression due to persistent hyperglycemia and aging, which is expressed in a lower quality of life.

## 1. Introduction

Diabetes mellitus (DM) is a chronic disease that requires constant care and monitoring and is accompanied by multiple complications, quality of life impairment, and decreasing life expectancy. Managing patients with DM is complex; it requires a multifactorial and multidisciplinary approach that is individualized and centered on the patient. More than half a billion people live with DM worldwide (537 million), affecting men, women, and children of all ages in every country, and this number is expected to double in the next 30 years. Recent estimates published in The Lancet say that in 2050, the number of people with DM will be 1.3 billion. Worldwide, the estimated prevalence of type 2 DM (T2DM) is between 6% and 9%; 90–95% of cases are T2DM. Increased body weight is the main risk factor for T2DM, followed by dietary behavior, environmental/occupational risks, smoking, low physical activity, and alcohol use [1,2,3].

Worldwide, 1 in 8 people, or 970 million people, have a mental disorder (significant disturbances in thinking, emotional regulation, or behavior). Typically, a mental disorder is characterized by distress or impairment across a range of functional domains. Anxiety and depression disorders are the most common [4,5,6]. In 2020, the number of people with anxiety and depression has increased significantly due to the COVID-19 pandemic. Preliminary assessments indicate that anxiety and depressive disorders have increased by 26% and 28%, respectively, in one year. In Romania, the prevalence of mental and behavioral disorders has almost doubled in the last 10 years, reaching from 1566 cases/100,000 inhabitants in 2012 to 2890 cases/100,000 inhabitants in 2021. The prevalence of mental illnesses (which represents only a part of mental and behavioral disorders) has increased six times in the last 10 years, reaching from 2183 cases/100,000 in 2012 to 13,035 cases/100,000 in 2021 [7,8,9,10].

The relationship between psychiatric conditions and DM is bidirectional. The appearance of mental suffering in the DM patient will lead to a reserved prognosis for the appearance and worsening of complications [11,12]. The development of DM in a patient with a psychiatric disorder will lead to postponing the diabetes consultation as much as possible without following the appropriate treatment and the appearance of complications [13,14,15]. The diagnosis of DM is always a major stress factor for the patient because it requires radical changes in their daily life, with the need to manage a complex treatment with constant attention to a new diet, blood sugar, and medication that will be necessary for their whole life [16,17,18].

T2DM, especially accompanied by obesity, is more frequently associated with micronutrient deficiencies (vitamins and minerals, such as vitamin D, group B vitamins, and antioxidants) that play a role in supporting the immune system and are involved in a series of reactions in the immune system [19,20]. Poor glycemic control with high glycemic variability and numerous complications and comorbidities in patients with DM decreases the ability to defend against infections, including viral infections, such as infection with the new coronavirus (SARS-CoV-2). Chronic inflammation is exacerbated during infection with the new coronavirus, leading to severe complications, and micronutrient deficiencies are associated with increased virulence and negative prognosis [21,22,23,24].

The main objective of our study was to evaluate the psychiatric impairment in patients with T2DM, especially anxiety–depressive disorders, as well as the assessment of cognitive decline and the relationship between them. We evaluated the main cardiovascular risk factors and quality of life, looking for possible correlations with psychiatric impairment.

## 2. Materials and Methods

### 2.1. Study Design and Patients

The patients were selected from 689 admitted to the Internal Medicine-Diabetes, Nutrition, and Metabolic Diseases Clinic of the “Pius Brinzeu” County Emergency Hospital Timisoara between January 2024 and April 2024. Following the inclusion criteria (T2DM, Hb > 7 g/dL, BMI ≥ 18 kg/m^2^, age > 18 years), this study included 209 T2DM patients in a cross-sectional manner, with a median age of 66.0 (58; 70) years and a median DM duration of 9 (6; 15) years. The patients were previously known to have T2DM and were discharged periodically (at intervals of 3 to 6 months, respectively) through the Diabetes Center. Patients were of average economic status, either employed or retired, with no significant family background problems. The research was conducted under the Declaration of Helsinki (2013 version). All patients signed the informed consent. The Ethics Committee of Timișoara County Emergency Hospital approved the study protocol (416/15 November 2023). The exclusion criteria were age < 18 years, BMI < 18 kg/m^2^, a severe form of anemia (Hb ≤ 7 g/dL), severe liver and kidney damage, neurological conditions, or other psychiatric conditions.

### 2.2. Patients Medical Assessments

This study assessed many patient data, including sex, age, and duration of DM, and anthropometric indexes such as weight, height, and body mass index (BMI). The individuals were interrogated regarding their alcohol use, smoking habits, and level of physical exercise. In addition, other biological parameters were assessed, including lipid panel and inflammatory indicators such as erythrocyte sedimentation rate (ESR), C-reactive protein (CRP), and fibrinogen. In order to assess renal function, the estimated glomerular filtration rate (eGFR) and urine albumin/creatinine ratio (UACr) were measured. Fasting glycemia (FG), postprandial glycemia (PPG), and glycated hemoglobin (HbA1c) were utilized to evaluate glycemic control.

To establish the diagnosis of anxiety/depression, we used questionnaires, and to assess the cognitive status, we performed the Mini-Mental State Examination (MMSE).

In our investigation, we utilized the Hospital Anxiety and Depression Scale (HADS), a measurement tool developed by Zigmond and Snaith in 1983. The primary objective of this tool is to offer doctors a practical instrument that is deemed acceptable, trustworthy, valid, and user-friendly, enabling them to identify and quantify symptoms of depression and anxiety effectively. The scale serves a dimensional purpose rather than a category one. It is most effective in identifying general hospital patients who require additional psychiatric examination and support rather than making diagnoses of psychiatric diseases. The interpretation of the total score of HADS is the following: Depression (D)/Anxiety (A): 0–7 = Normal, 8–10 = Borderline abnormal (borderline case), mild, 11–14 = Moderate, 15–21 = Severe [25,26].

The EQ-5D-5L is a self-evaluated questionnaire that measures health-related aspects of quality of life. The scale assesses the quality of life using a five-component framework, encompassing mobility, self-care, engagement in routine activities, pain and distress, and anxiety and depression. Each level is assessed using a scale that quantifies the extent of difficulties in that particular domain (e.g., I have no difficulties walking around, minor, moderate, significant, or complete inability to walk). The tool additionally has a comprehensive health scale, wherein the rater assigns a numerical value ranging from 1 to 100 to characterize their health status, with 100 representing the highest conceivable state [27,28].

(MMSE) is a psychometric tool utilized to evaluate an individual’s mental state. The Mini-Mental State Examination (MMSE) was initially introduced by Folstein in 1975 as a tool for the timely identification and surveillance of dementia. The examination assesses memory, registration, attention, and language abilities. The MMSE categorizes impairment levels as follows: 27 at Normal, 21–26 at Mild, 11–20 at Moderate, and 11 at Severe [29,30,31].

### 2.3. Statistical Analysis

The statistical analysis was conducted using MedCalc^®^ Statistical Software version 22.016, developed by MedCalc Software Ltd. in Ostend, Belgium. The software may be accessed at https://www.medcalc.org and was used on 4 December 2023. The mean and standard deviation, median, and minimum and maximum values were used to depict the continuous variables according to their distribution. The presentation of categorical variables included both absolute values and percentages. For the comparison of two sets of continuous, non-normal distributed variables, the Mann–Whitney test was applied. The *t*-test was applied to compare two continuous, normally distributed variable sets. The variation in median values among more than two groups was analyzed using the Kruskal–Wallis test. In order to investigate the strength and direction of the correlations among the numerical variables, a correlation analysis was performed, followed by multivariate and logistic regression analysis. To assess the predictive capability of prospective factors with respect to a continuous variable’s value, “Receiver-Operating Characteristics” (ROC) analyses were conducted. We provided the 95% confidence interval for the statistical analyses and deemed a *p*-value below 0.05 to be statistically significant.

## 3. Results

In the final analysis, 209 patients with T2DM were included, of which 34.93% were men (73/209) and 65.07% were women (136/209). The median age of the patients was 66.0 (58; 70) years, and the median duration of DM was 9 (6; 15) years. The patients exhibited suboptimal glycemic control, evidenced by a median HbA1c 7.8 (7; 9.2) mg/dL (Table 1).

No significant differences were observed between the two genders, except for weight, which was higher in men than in women: 88.15 ± 12.50 kg, 79.75 ± 14.03, and eGFR (*p* < 0.0001, Table 1), respectively.

Over half of the patients with T2DM who participated in the research exhibited diverse manifestations of anxiety or depression. Only two patients (0.9%) had severe anxiety, while 67 patients (32.1%) had mild anxiety, and 39 patients (18.7%) had moderate anxiety. Of the total number of patients, 101 (48.3%) had no anxiety. We discovered that 89 patients (42.5%) did not report depressive states, 67 patients (32.1%) had mild depression, 48 patients (23%) had moderate depression, and 5 patients (2.4%) had a severe form of depression in response to the questionnaire regarding depression. A comparison was made between the primary investigated parameters based on levels of anxiety and depression (Table 2).

There was a direct and positive correlation observed between the anxiety score (HADS) and other factors, including age, duration of DM, HbA1c levels, FG, PPG, and depression score. A moderate and unfavorable association has been seen between the anxiety score and both cognitive status and quality of life (Table 3).

Similarly, the depression score had a better and positive association with patient age and poor glycemic control. The depression score negatively impacts the cognitive status and quality of life, reflected by the correlation coefficient in Table 4.

In a multivariate regression analysis, we identified an association between anxiety score and age, PPG, depression score, and quality of life. The characteristics of the regression models are presented in Table 5.

We constructed logistic regression models to determine the effects of different parameters correlated with the anxiety score HADS (age, DM duration, FG, and PPG) in patients with T2DM on the probability of developing anxiety or depression. In the univariate logistic regression models, we identified age as a significant risk factor for development anxiety (OR = 1.035, 95% CI: 1.0039–1.685, *p* = 0.02). Also, the PPG is an important factor that causes anxiety (OR = 1.0231, 95% CI: 1.0114–1.0350, *p* < 0.0001).

**Figure 1:**
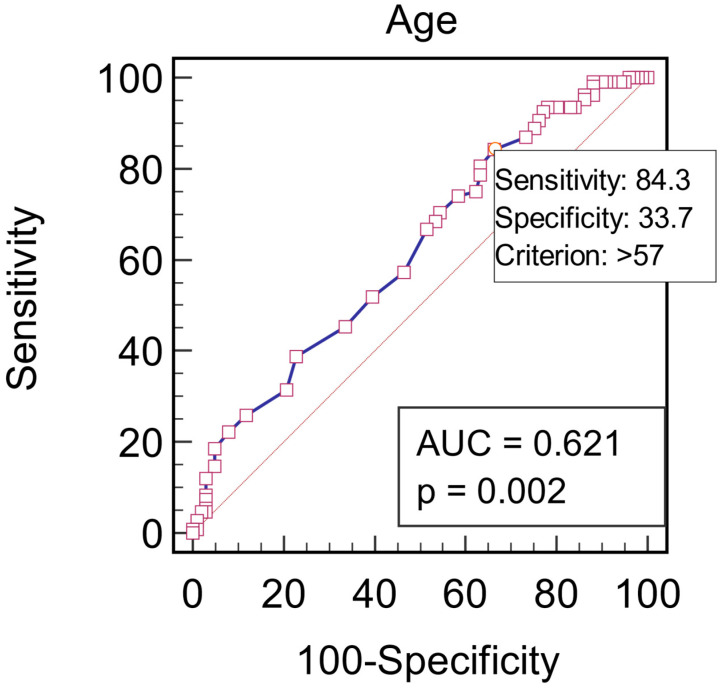
Graphical representation of the ROC curve of the age for the prediction of anxiety.

We also constructed ROC curve models to evaluate the possibility of predicting anxiety and depression. According to the ROC curve (AUROC = 0.621, *p* = 0.002), age > 57 represents a statistically significant predictive factor of anxiety, with a sensitivity of 84.3 and specificity of 33.7 (Figure 1).

**Figure 2:**
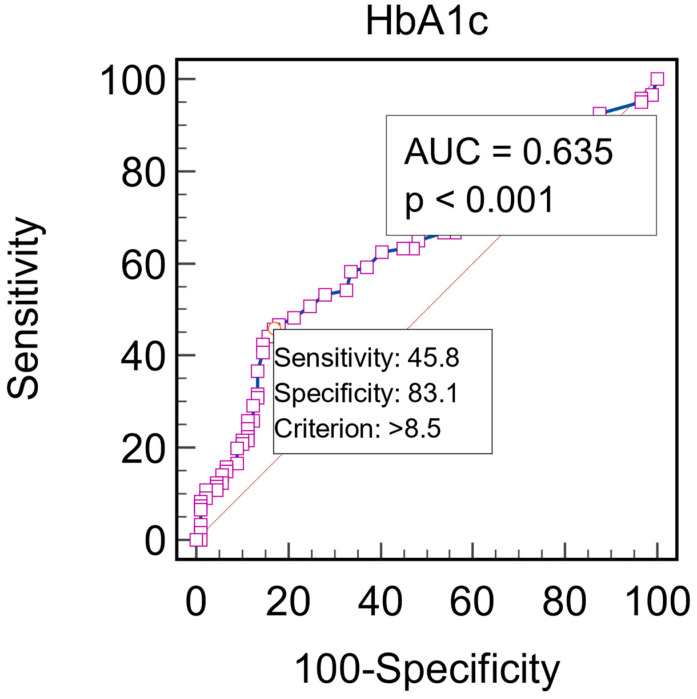
Graphical representation of the ROC curve of the HbA1c for the prediction of depression.

Deriving univariate logistic regression models, we identify as a significant risk factor for depression the duration of DM (OR = 1.1004, 95% CI: 1.0439–1.1599, *p* = 0.0004) and HbA1c (OR = 1.12769, 95% CI: 1.0548–1.15458, *p* = 0.01). An HbA1c >8.5% represents a statistically significant predictive factor of depression, with a sensitivity of 45.8 and specificity of 83.1, according to the ROC curve (AUROC = 0.635, *p* < 0.0001) presented in Figure 2.

## 4. Discussion

This present research analyzed the probability of developing anxiety or depression in patients living with T2DM during their lifetime. We found that 51.7% of them were affected by anxiety, and 37.5% of them were also living with depression.

Psychiatric disorders, especially depression, were studied more frequently in patients with T2DM as a predictive factor, as well as coexisting conditions. Numerous studies have shown that depression causes the appearance of T2DM, with these patients having over 60% risk of DM [32,33].

One of the biggest challenges in the management of psychiatric disorders associated with DM is their low detection rate: 45% of cases of mental illness remain undetected in patients with DM. The distress generated by DM occurs because patients are in a situation that completely changes their life, forcing them to develop complex behaviors to manage their blood sugar [34,35,36].

The burden of the disease and the need to adapt to the new lifestyle produce great emotional suffering. At the time of diagnosis, each patient goes through the healing stages of shock:

- the first that appears is DENIAL—they cannot believe that it is happening to them and do not want to accept, hear, or have contact with what the disease means;
- the next stage is ANGER—the patient becomes angry at the whole situation and the people around them;
- then comes the DEPRESSION phase, in which the patient becomes sad, it seems unfair that this is happening to them, and they ask themselves questions like “Why me?”;
- finally, there is ACCEPTANCE—the patient realizes and accepts the situation [37,38,39].

Among the mood disorders in DM patients, the most common is depressive disorder, which is associated with the alteration of glycemic control and increased mortality. The risk of developing depression in the diabetic population is over 50% higher compared to the general population [40,41,42].

A series of similar biochemical changes are associated with the two medical conditions, such as stress, which activates the hypothalamic–pituitary axis, increases in cortisol, alteration of glucose transport, and the presence of pro-inflammatory cytokines. Thus, the interdependence of the two diseases, with the amplification of physiopathological pathways and the alteration of mental health, is determined [43,44,45].

The symptoms of depression are complex and include

- feelings of sadness and inner emptiness, irritability, and guilt;
- sleep problems (patients cannot sleep or, on the contrary, sleep too much);
- overeating or not eating at all;
- fatigue, difficulty concentrating and making decisions;
- lack of hope; and
- thoughts of death or suicidal ideation [46,47,48].

Depression with psychotic aspects and the manifestation of delirium, encompassing both hyperactive and hypoactive clinical manifestations, are among the most undesirable consequences experienced by individuals with DM. Prompt detection of delirium symptoms is crucial, as it is linked to a significant mortality risk. Individuals experiencing delirium exhibit symptoms of confusion and disorientation. In cases of hyperactive delirium, individuals display agitation and engage in irrelevant and uncoordinated movements. Conversely, hypoactive delirium is characterized by tranquility and diminished motor activity. Episodes of hypoglycemia or diabetic ketoacidosis have the potential to induce delirium. Therefore, it is imperative to promptly rectify these issues through vigilant monitoring and supportive therapy, including the administration of modest dosages of antipsychotics (such as haloperidol) for behavioral management [49,50,51].

Anxiety is the second psychiatric disorder commonly observed in individuals with DM, often manifesting in conjunction with other consequences. Needle phobia and hypoglycemia phobia are two anxiety disorders that are commonly linked to DM. Uncontrolled blood sugar levels, increased HbA1c, and the development of other long-term complications are observed due to the association between these two types of phobias, as well as the clinical superposition of hypoglycemia with anxiety symptoms (hand tremors, confusion, sweating, tachycardia) [52,53,54,55].

In addition to depression and anxiety disorders, individuals with DM may also have other psychiatric illnesses, such as obsessive–compulsive disorder, which is characterized by excessive monitoring of blood glucose levels. There exists a correlation between schizophrenia and diminished glucose tolerance as well as insulin resistance. It has been established that individuals diagnosed with schizophrenia exhibit a 2–4-fold increased susceptibility to acquiring DM in comparison to the broader community. Alcohol misuse is the most prevalent addiction issue among patients with DM, with a prevalence rate of 50–60% [56,57,58].

The prevalence of depression was found to be 56.9% in males and 43.1% in women, according to a study conducted by Alzahrani and colleagues with 450 diabetic patients. This study found that age, gender, HbA1c, presence of comorbidities, and duration of DM were all predictors of depression [59]. According to the findings of Khullar et al., the prevalence of depression in women was found to be greater than in males (49.87%), and the researchers also discovered that female gender, duration of DM, and BMI were independent risk factors for depression [60]. Similarly, in our research, we found that age, the duration of T2DM, and poor glycemic control were all predictive indicators for anxiety and depression in people who had T2DM.

An increase in self-efficacy as a sanogenic personality attribute can be achieved through patient education in self-management, focusing on attitude development. The introduction of interventions that were based on educational programs to develop in these patients the concept of self-management of the disease and the prevention of complications through assertive training to improve DM control behavior was found to result in a significant improvement in the quality of life of patients who were diagnosed with DM [61,62]. It has been demonstrated that there is a correlation between depression and macrovascular damage. This association is independent of the level of HbA1c and serum lipids, suggesting that other mechanisms are involved in coronary and peripheral arterial disease. These mechanisms were found to be correlated with the MADRS (Montgomery Åsberg Depression Rating Scale) score, a scale for the evaluation of depressive symptoms. For the first time, the MADRS was applied to patients who were diagnosed with major depression in order to evaluate changes in the severity of depression. The MADRS is a questionnaire that consists of ten questions; each scenario that is described has many options, and each answer has the potential to receive anywhere from 0 to 6 points [63,64,65].

Given that weight gain is a side effect of antidepressants, there is a pressing need for enhanced attention to be paid to the use of these medications in diabetic patients from a therapeutic perspective. Therefore, it is recommended that blood glucose levels (both fasting and postprandial glucose) and HbA1c be evaluated regularly after meals. When it comes to the management of mild forms of depression and anxiety among high-risk groups, such as pregnant women and children, it is recommended that psychotherapy or counseling be utilized rather than antidepressant medication [66,67,68].

According to Chen et al., sedentarism, obesity, and the presence of comorbidities are factors that increase the risk of depression in older individuals with T2DM [69]. It was found that the majority of people who were diagnosed with DM experienced at least one complication that was connected to their age, gender, and socioeconomic status [70]. More than 50% of the patients included in our study presented a form of anxiety or depression.

Nevertheless, the current investigation is constrained by other aspects, including a relatively small sample size of patients examined. Furthermore, the current study design is insufficient in evaluating the causal link between depression, anxiety, cognitive state, and T2DM. Another limitation is that the study was performed on patients admitted to the hospital for different medical reasons. Therefore, their level of depression and anxiety could be higher than in outpatients. Subsequent extensive investigations employing case-control designs could clarify the correlation between psychiatric problems and the impact of T2DM.

## 5. Conclusions

The risk of anxiety/depression is directly proportional to age, duration of DM, and glycemic control. Patients with T2DM, aged over 57 years, with an HbA1c> 8.5%, have a significantly higher risk of developing anxiety/depression during their lifetime. Poor adherence to treatment and the connection between DM and a variety of psychiatric conditions will result in a substantial decline in quality of life and inadequate glycemic control. Additionally, it results in a rise in hospitalizations and expenses. Although DM is incurable, emotional, mental, and behavioral healing can improve prognoses.

## Figures and Tables

**Table 1:** The characteristics of the patients included in our study.

Variable	Overall	Men	Women	*p*
Age (years) ^a^	66.0 (58; 70)	65 (101.2)	66 (106.9)	0.51
DM duration (years) ^a^	9.0 (6; 15)	9 (99.9)	9.5 (107.7)	0.37
Weight (kg) ^b^	82.7 ± 14.1	88.1 ± 12.5	79 ± 14	<0.0001
BMI (kg/m^2^) ^a^	29 (26; 33)	29 (99.5)	29 (107.9)	0.34
FG (mg/dL) ^a^	135 (122.7; 150)	138 (113.2)	132.5 (100.5)	0.14
PPG (mg/dL) ^a^	155 (140; 178)	154 (109.4)	156 (102.6)	0.43
HbA1c (%) ^a^	7.8 (7; 9.2)	7.9 (107.4)	7.8 (103.6)	0.66
TC (mg/dL) ^a^	181 (49; 342)	182 (108.5)	178.5 (103)	0.5
TG (mg/dL) ^a^	148 (47; 469)	141 (101.6)	155 (106.8)	0.5
LDLc (mg/dL) ^a^	94 (31; 204)	95 (106.8)	93.5 (103.9)	0.7
HDLc (mg/dL) ^a^	43 (23.5; 87)	44 (110.4)	43 (102)	0.3
eGFR (mL/min) ^b^	81.8 ± 26.4	89.4 ± 26.2	77.8 ± 25.7	0.002
UACr (mg/g) ^a^	35 (3; 756)	35 (105.8)	34.1 (104.5)	0.8
ESR (mm at 1 h) ^a^	18 (12; 33)	16 (96.56)	20 (109.5)	0.13
CRP (mg/L) ^a^	11 (6; 26)	8.9 (97.8)	12 (108.8)	0.20
Fibrinogen (mg/dL) ^a^	334 (278; 426)	312 (94.3)	354 (110.6)	0.06
Anxiety Score (HADS) ^a^	7 (5; 9)	8 (109.3)	7 (102.6)	0.44
Depression Score (HADS) ^a^	8 (4; 11)	8 (112.5)	8 (100.9)	0.18
Cognitive Status (MMSE) ^a^	24 (22; 25)	23 (99.3)	24 (108)	0.32
Quality of Life (EQ-5D-5L) ^a^	60 (45; 70)	55 (104.6)	60 (105.1)	0.95

Abbreviations: DM, diabetes mellitus; BMI, body mass index; FG, fasting glycemia; PPG, postprandial glycemia; HbA1c, glycated hemoglobin; ESR, erythrocytes sedimentation rate; CRP, C-reactive protein, Hospital Anxiety, and Depression Scale (HADS); Mini-Mental State Examination (MMSE). ^a^ Continuous variables (with non-Gaussian distribution) are indicated by their median (interquartile range in men and women, or minimum–maximum values overall). ^b^ Continuous variables (with Gaussian distribution) are indicated by their mean (standard deviation).

**Table 2:** Characteristics of the patients according to anxiety levels and depression severity.

Psychiatric Disorder	Age (Years)	DM Duration (Years)	HbA1c (%)	FG (mg/dL)	PPG (mg/dL)
No anxiety	65 (54.7; 68)	8 (5; 14)	7.3 (6.8; 8.3)	131 (118; 143)	147 (133.7; 162)
Mild Anxiety	67 (60; 70)	9 (7; 12.7)	8.3 (7.3; 9.6)	142 (128; 151.7)	162 (148.2; 186)
Moderate Anxiety	67 (62.5; 71.5)	14 (7.2; 16.7)	8.4 (7.1; 9.3)	141 (125; 161.7)	175 (148.2; 200.2)
Severe Anxiety	72.5 (71; 74)	16.5 (13; 20)	7.4 (6.4; 8.4)	118.5 (86; 151)	169.5 (140; 199)
p *	0.0001	0.01	0.001	0.0006	<0.0001
No Depression	63 (52.7; 69)	8 (4.7; 12)	7.3 (6.9; 8.2)	132 (121; 144)	147 (134.7; 158.5)
Mild Depression	66 (58.2; 68)	9 (6; 13)	8.4 (7.1; 9.5)	129 (121.2; 144)	153 (141; 178.2)
Moderate Depression	69 (65; 73.5)	14.5 (10.5; 20)	9.6 (8.9; 10.2)	151 (134; 162)	186 (162; 202)
Severe Depression	70 (65.7; 75.2)	14 (11.2; 21.2)	9.4 (8.2; 10.5)	148 (139; 161.7)	210 (177.7; 221.7)
p *	0.0001	<0.0001	<0.0001	<0.0001	<0.0001

* Kruskal–Wallis test. Abbreviations: DM, diabetes mellitus; HbA1c, glycated hemoglobin; FG. fasting glycemia; PPG, postprandial glycemia. Continuous variables (with non-Gaussian distribution) are indicated by their median (minimum–maximum values).

**Table 3:** Correlations between anxiety score (HADS) and studied parameters.

Parameter	Correlation Coefficient r	95% Confidence Interval for r	*p*
Age (years)	0.2461	0.1142–0.3695	0.0003
DM duration (years)	0.1756	0.04088–0.3041	0.0110
HbA1c (%)	0.1336	−0.002119–0.2646	0.05
FG (mg/dL)	0.2747	0.1443–0.3956	0.0001
PPG (mg/dL)	0.2975	0.1686–0.4164	<0.0001
Depression Score (HADS)	0.5359	0.4316–0.6261	<0.0001
Cognitive status (MMSE)	−0.4336	−0.5377–−0.3165	<0.0001
Quality of life (EQ-5D-5L)	−0.5511	−0.6390–−0.4489	<0.0001

Abbreviations: DM, diabetes mellitus; HbA1c, glycated hemoglobin; FG, fasting glycemia; PPG, postprandial glycemia; Hospital Anxiety and Depression Scale (HADS); Mini-Mental State Examination (MMSE).

**Table 4:** Correlations of the depression score (HADS) and studied parameters.

Parameter	Correlation Coefficient r	95% Confidence Interval for r	*p*
Age (years)	0.4127	0.2934–0.5193	<0.0001
DM duration (years)	0.1756	0.04088–0.3041	0.0110
HbA1c (%)	0.3091	0.1810–0.4270	<0.0001
FG (mg/dL)	0.3835	0.2614–0.4936	<0.0001
PPG (mg/dL)	0.4927	0.3826–0.5891	<0.0001
Anxiety Score (HADS)	0.5359	0.4316–0.6261	<0.0001
Cognitive status (MMSE)	−0.5768	−0.6608–−0.4785	<0.0001
Quality of life (EQ-5D-5L)	−0.7521	−0.8056–−0.6864	<0.0001

Abbreviations: DM, diabetes mellitus; HbA1c, glycated hemoglobin; FG, fasting glycemia; PPG, postprandial glycemia; Hospital Anxiety and Depression Scale (HADS); Mini-Mental State Examination (MMSE).

**Table 5:** Multivariate regression analysis for factors associated with anxiety and depression.

Independent Variables	Coefficient	Std. Error	t	*p*	r_partial_	r_semipartial_
Anxiety score (HADS) *p* = 0.004 for model, multiple correlation coefficient = 0.34						
Age (years)	0.05512	0.02048	2.691	0.0077	0.1843	0.1759
PPG (mg/dL)	0.02437	0.006567	3.711	0.0003	0.2503	0.2426
Anxiety score (HADS) *p* < 0.0001, for model, multiple correlation coefficient = 0.58						
Depression score (HADS)	0.2372	0.07295	3.251	0.0013	0.2209	0.1843
Quality of life (EQ-5D-5L)	−0.06278	0.01585	−3.961	0.0001	−0.2660	0.2246
Depression score (HADS), *p* < 0.0001, for model, multiple correlation coefficient = 0.57						
Age (years)	0.1096	0.02106	5.203	<0.0001	0.3408	0.2966
PPG (mg/dL)	0.04745	0.006752	7.027	<0.0001	0.4397	0.4005
Depression score (HADS), *p* < 0.0001, for model, multiple correlation coefficient = 0.76						
Anxiety score (HADS)	0.2058	0.06329	3.251	0.0013	0.2209	0.1456
Quality of life (EQ-5D-5L)	−0.1425	0.01166	−12.22	<0.0001	−0.6483	0.5473

Abbreviations: PPG, postprandial glycemia; Hospital Anxiety and Depression Scale (HADS).

## Data Availability

Patients who participated in this study did not provide written consent for the public dissemination of their data; therefore, supporting data are unavailable due to the sensitive nature of the research.
